# Evaluation of the Susceptibility of the Pea Aphid, *Acyrthosiphon pisum*, to a Selection of Novel Biorational Insecticides using an Artificial Diet

**DOI:** 10.1673/031.009.6501

**Published:** 2009-12-03

**Authors:** Amin Sadeghi, Els J.M. Van Damme, Guy Smagghe

**Affiliations:** ^1^ Department of Crop Protection, Faculty of Bioscience Engineering, Ghent University, Coupure Links 653, 9000 Ghent, Belgium; ^2^ Department of Molecular Biology, Faculty of Bioscience Engineering, Ghent University, Coupure Links 653, 9000 Ghent, Belgium

**Keywords:** artificial diet bioassay, aphid, flonicamid, pymetrozine, imidacloprid, flufenoxuron, pyriproxyfen, Neem Azal-T/S, halofenozide, toxicity, nymphal survival

## Abstract

An improved technique was developed to assay the toxicity of insecticides against aphids using an artificial diet. The susceptibility of the pea aphid *Acyrthosiphon pisum* (Harris) (Hemiptera: Aphidoidea) was determined for a selection of novel biorational insecticides, each representing a novel mode of action. Flonicamid, a novel systemic insecticide with selective activity as feeding blocker against sucking insects, showed high toxicity against first-instar *A. pisum* nymphs with an LC_50_ of 20.4 μg/ml after 24 h, and of 0.24 µg/ml after 72 h. The toxicity was compared with another feeding blocker, pymetrozine, and the neonicotinoid, imidacloprid. In addition, four insect growth regulators were tested. The chitin synthesis inhibitor flufenoxuron, the juvenile hormone analogue pyriproxyfen, and the azadirachtin compound Neem Azal-T/S showed strong effects and reduced the aphid population by 50% after 3 days of treatment at a concentration of 7–9 µg/ml. The ecdysone agonist tested, halofenozide, was less potent. In conclusion, the improved aphid feeding apparatus can be useful as a miniature screening device for insecticides against different aphid pests. The present study demonstrated rapid and strong toxicity of flonicamid, and other biorational insecticides towards *A. pisum.*

## Introduction

Aphids (Homoptera) are one of the most important groups of insect pests in the world. To date, about 4,000 aphid species have been described, and about 250 species are serious pests to various crops and ornamental plants around the world. Aphids develop at prodigious rates by parthenogenesis and have an efficient dispersal strategy. Their feeding of phloem sap causes stunting, discoloration and deformation of plants, and aphids are major vectors of plant viruses. Although many products belonging to existing insecticide groups are effective against aphids, resistance to insecticides that have a long history of use, such as organophosphates, carbamates and pyrethroids, is a serious problem to farmers and the environment, beneficial insects and natural enemies (Dixon 1985; Sylvester 1987; Blackman and Eastop 2000; Després et al. 2007). Due to the known harmful effects of such conventional pesticides, there is a growing use of pesticide alternatives to reduce risks. Alternatives are currently being investigated and include the use of biorational compounds that are compatible with integrated pest management (Horowitz and Ishaaya 2004). According to the US-Environmental Protection Agency biorational pesticides pose minimal risk to the environment, degrade quickly, leave minimal residue, are safe to handle, and relatively small quantities are required for effective control. Pesticides classified as biorational include various classes of insect growth regulators (IGRs), microbial products, synthetic molecules with novel modes of action and plant-derived compounds.

Flonicamid and pymetrozine are two novel insecticides with selective activity against Homoptera, acting as feeding inhibitors with high mortality due to starvation (Harrewijn and Kayser 1997; Denholm et al. 1998; Morita et al. 2007). Imidacloprid is the most important neonicotinoid insecticide with good systemic activity that acts as an agonist of the insect nicotinyl acetylcholine receptors, causing the insect to reduce or stop feeding and mobility. It is particularly effective against aphids, whiteflies and planthoppers (Boiteau and Osborn 1997; Elbert et al. 1998; Nauen et al. 1998).

IGRs are novel insecticides that interfere in the processes of molting and metamorphosis of insects. Two major insect-specific target processes are the biosynthesis of chitin in cuticle and the activity of hormones such as juvenile hormone and the insect molting hormone, 20-hydroxyecdysone. Over the last decades several IGRs have been developed such as chitin synthesis inhibitors (e.g., diflubenzuron and flufenoxuron), juvenile hormone analogues (e.g., pyriproxyfen), ecdysone agonists (e.g., RH-5849 and halofenozide) and azadirachtin-based products (e.g., Neem Azal T/S). Some IGRs are active against aphids as reported by Hatakoshi et al. (1991) and Kerns and Stewart (2000).

The pea aphid *Acyrthosiphon pisum* (Harris) (Hemiptera: Aphidoidea) was selected for this study as this aphid is responsible for hundreds of millions of dollars of crop damage every year, and it is one of the primary aphid species used in the laboratory. Hence many populations have already acquired resistance towards conventional pesticides. The objectives of the present study were to improve a technique to bioassay the toxicity of various insecticides on aphids using an artificial diet. The compunds tested included the feeding blockers, flonicamid, pymetrozine, and imidacloprid, and four insect growth regulators flufenoxuron, pyriproxyfen, Neem Azal-T/S and halofenozide.

Incorporating the chemical into the food source is a standard technique for evaluating chemicals towards insects. However, one of the problems encountered in studies involving the effect of insecticides on sap-sucking insects such as aphids is the problem of administering them via feeding to these insects. Many attempts have been made to rear aphids on artificial diets (Mittler and Dadd 1964; Auclair 1965; Febvay et al. 1988). The use of an artificial diet allows easy testing of small quantities of synthetic compounds and challenging aphids to oral exposure under controlled conditions. In addition, this technique is simple, fast, and inexpensive and is especially suitable for short-term studies involving the effects of toxins on aphids. It may also be used to study the effects of growth factors, hormones and special nutrients on aphid growth and possibly on other sucking insects. The overall concept was to make the bioassay miniature, easy-to-handle and standardized. Using this technique the potency of a selection of novel biorational insecticides was evaluated.

## Materials and Methods

### Insect

The pea aphid *A. pisum* clone was initially brought to our laboratory from a culture at Biobest NV (Westerlo, Belgium). All stages of the aphid are maintained on young broad bean, *Vicia faba* L. (Fabales: Fabaceae), plants under standard conditions of 25 ± 5° C, 65 ± 5% relative humidity and a photoperiod of 16 h light. Mature aphids were put on plants for 24 h, resulting in neonate nymphs with an age of 0–24 h that were used throughout the experiments.

### Insecticides

The following commercial formulations of seven insecticides were evaluated: flonicamid (Teppeki®, 50WG, 500 g AI per kg; Ishihara Sangyo Kaisha Ltd., www.iskweb.co.jp/), pymetrozine (Chess®, 25WP, 250 g AI per kg; Syngenta AG, www.syngenta.com), imidacloprid (Confidor®, 200SL, 200 g AI per liter; Bayer Cropscience, www.bayercropscience.com), flufenoxuron (Cascade®, 10EC, 100 g AI per liter; BASF AG, www.basf.com), pyriproxyfen (Admiral®, 10EC, 100 g AI per liter; Sumitomo Chemical, www.sumitomochem.co.jp), halofenozide (RH-0345, 240 g AI per liter; Rohm and Haas, www.rohmhaas.com), and Neem AzalT/S (1% azadirachtin; Trifolio-M GmbH, www.trifoliom.de).

**Figure 1.**
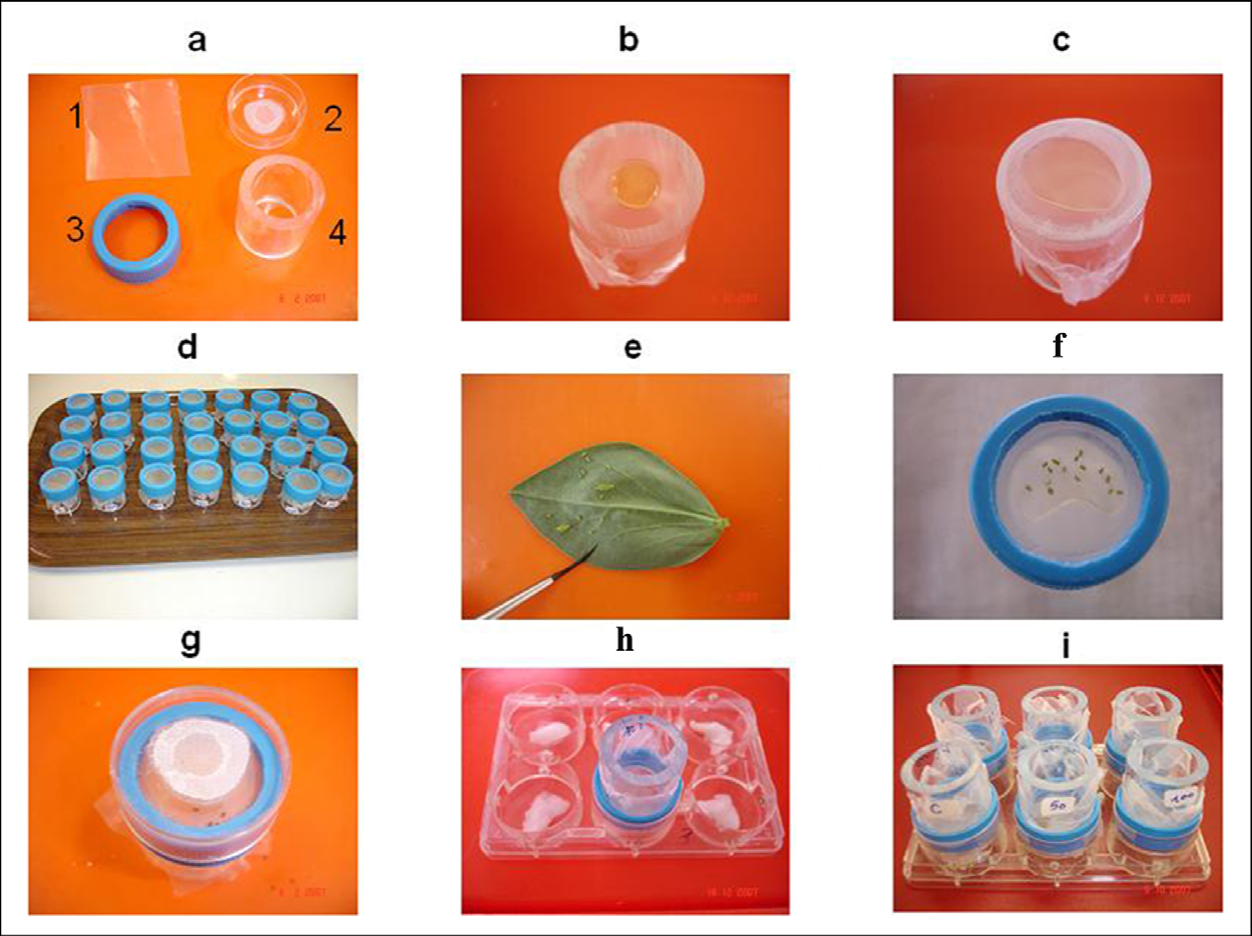
Scheme to prepare the aphid feeding apparatus showing component parts.

### Artificial diet

A standard diet previously developed for *A. pisum* (Febvay et al., 1988) was used as the basal diet to which the test compounds were added. The prepared liquid artificial diet was filter-sterilized through a 0.2 µm filter (FP 30/ 0,2 CA-S, Schleicher and Schuell, www.s-and-s.com). Aliquots of 10 ml could be stored in the freezer at -20°C for a period up to 6 months.

### Aphid feeding apparatus

**Figure 2.**
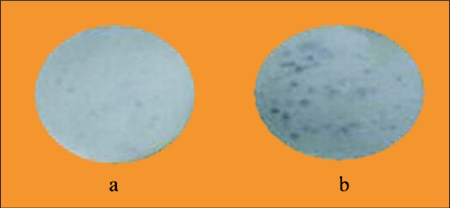
Treatment of *Acyrthosiphon pisum* during 24 h with flonicamid at 100 µg/ml (a) in the artificial diet reduced markedly the amounts of honeydew produced by aphid nymphs as compared to the untreated control (b) after visualization by use of the Ninhydrin test.

**Figure 3.**
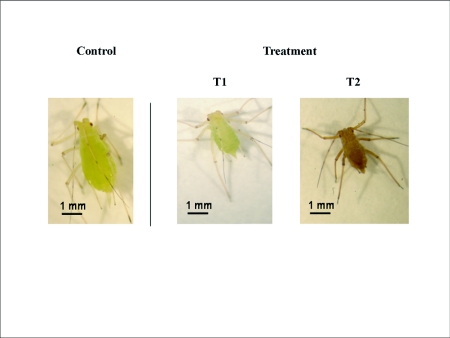
First-instar nymphs of *Acyrthosiphon pisum* treated for 48 h with 100 µg/ml flonicamid. In intoxicated aphids, clear symptoms were observed of inhibited nymphal growth as compared to control aphids (Control) with a significant reduction in aphid size (Tl: the aphid size was reduced about 50%) and then followed by aphid mortality (T2: the dead aphid turns brown). With pymetrozine similar inhibitory effects and mortality were observed.

The feeding apparatus was constructed using a plexiglass ring (Figure 1a–4), a piece of Parafilm (Figure 1a–1), a rubber ring (Figure 1a-3) and a small Petri dish (Figure 1a–2). The food sachet was made under sterile conditions by slightly depressing a piece of Parafilm membrane (sterilized with 75% ethanol), on top of the plexiglass ring (h = 4 cm, Ø = 3 cm). Subsequently 200 µl of the artificial diet was pipetted on the membrane (Figure 1b) and covered with another piece of Parafilm membrane that was stretched 4 times (Figure 1c). All air was removed from the sachet. The edges of the two Parafilm membranes were then pressed firmly together against the plexiglass ring, and a strip of Parafilm membrane was placed over the sealed edges of the sachet around the edge of the plastic ring. Finally, a rubber ring (h=1.2 cm, Ø=3.4 cm) was placed over the sachet around the edge of the plexiglass ring (Figure 1d). After transfer of the aphids from the *V.faba* plants onto the sachet (Figure 1e) using a camel brush, a small Petri dish (h=1 cm, Ø=3.6 cm) was put on top of it (Figure 1f). To ventilate the feeding apparatus a hole (Ø=1 cm) was made in the Petri dish that was covered with net cloth(Figure 1g). A 6-well assay plate (Figure 1h, i) was used as a base for the feeders. Ventilation for the aphids was provided by boring four small holes (Ø=3 mm) through the walls of each well of the plate and humidity was maintained by placing a piece of wet cotton at the bottom of each well (Figure 1i).

### Bioassay

At day 0, neonate nymphs (aged 0–24 h) were obtained from the synchronized population reared on *V. faba* plants, and then transferred to a freshly prepared diet sachet feeding apparatus. Mortality was scored 24, 48 and 72 h after feeding and dead insects removed. The sachets were replaced every two days.

For each insecticide, an appropriate stock concentration was prepared in distilled water and diluted in diet. Three replicates were performed for each insecticide concentration tested. To determine LC_50_ values, a pretest was performed with a wide range of concentrations ranging from 0.0001 to 100 µg/ml, and based on these, a minimum of 5 concentrations were used. In addition, a corresponding untreated control was used for each insecticide. A total of 270 aphids were tested per insecticide.

### Effect on honeydew production

The amounts of honeydew produced by the aphids in the treatments as compared to controls were measured using the Ninhydrin test as described by Kanrar et al. (2002). In brief, a 3.6 cm-diameter Petri dish, as described above in the feeding apparatus (Figure 1i), was lined with a Whatman No. 3 filter paper so that drops of honeydew would fall on it. This filter paper was removed after 24 h, and sprayed with 0.1% ninhydrin reagent to detect the presence of honeydew spots.

### Data analysis

The total mortality for each treatment was corrected according to Abbott's formula based on the mortality seen in the control groups. In all experiments, mortality in the control groups ranged between 0 and 15% with an average ± SE of 6 ± 2%. The results obtained were analyzed using non-linear sigmoid curve fitting, and the activity of each treatment was evaluated on the basis of dose-response concentrations (LC_50_ values and the corresponding 95% confidence interval) using Prism v4; the goodness of fit to the curve model was evaluated on the basis of *R^2^* values (GraphPad Software Inc., www.graphpad.com) (Mommaerts et al., 2006).

**Table 1.**
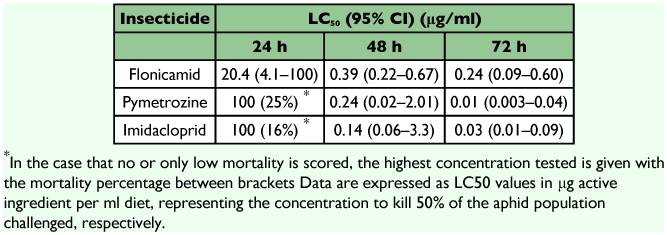
Acute toxicity of two feeding blockers flonicamid and pymetrozine, and the neonicotinoid imidacloprid against nymphs of the pea aphid *Acyrthosiphon pisum* when challenged for 24, 48 and 72 h in the artificial diet.

## Results

### Feeding inhibitors flonicamid, pymetrozine, and neonicotinoid imidacloprid

As shown in Table 1, the feeding inhibitor flonicamid showed a strong acute toxic activity with an LC_50_ value of 20.5 µg/ml in the first 24 h of treatment against aphids. Its activity increased and the LC_50_ value reached 0.24 µg/ml by day 3. Flonicamid treatment rapidly inhibited feeding by the aphids. Figure 2 shows a complete inhibition of honeydew spots in aphids treated with 100 µg/ml within the first 24 h compared to the aphids of the control groups. In addition, clear symptoms of inhibited nymphal growth were observed after 48 h; the size of aphids treated with 100 µg/ml flonicamid was reduced by 50% (Figure 3). There was no recovery of feeding during the experiment, and all aphids were dead after 72 h (Figure 3).

The other feeding inhibitor, pymetrozine, started with a lower activity, but gave a toxicity higher than flonicamid by day 3; the LC_50_ was 0.01 µg/ml. Similar symptoms of inhibition of feeding, reduced aphid size and no recovery were observed for pymetrozine as for flonicamid (Figure 3).

The neonicotinoid imidacloprid killed 16% of the aphids challenged with 100 µg/ml at day 1, and the LC_50_ was estimated at 0.03 µg/ml at day 3. Honeydew production was reduced at all concentrations tested after 24 h exposure. In treatments with the highest concentration very little honeydew was seen.

### IGR insecticides

The effect of the different IGRs tested towards neonates of *A. pisum* is shown in Table 2. Both Neem Azal-T/S and flufenoxuron had a similar toxicity after 3 days with respective LC_50_ of 7.9 and 8.7 µg/ml. Typical phenotypic symptoms of aphid mortality were disruption of nymphal molt and abortion of molting. With pyriproxyfen aphid susceptibility was somewhat lower with an LC_50_ of 9.3 µg/ml. With pyriproxyfen mortality before the first molt occurred as the aphids were smaller in size than in the controls and they died without molting. Halofenozide was at least 30 times less effective than the three other IGRs after 72 h exposure. The highest concentration tested, 100 µg/ml, resulted in 24% mortality and the LC_50_ was estimated as 304 µg/ml. Symptoms of halofenozide were inhibition of aphid size, growth and molting, resulting in mortality.

**Table 2.**
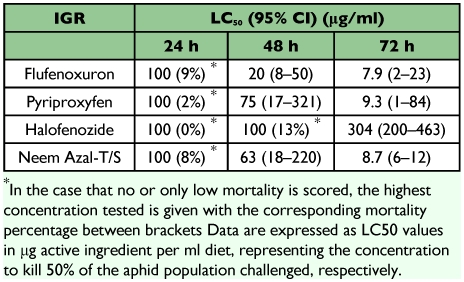
Acute toxicity of the four tested IGR insecticides against nymphs of the pea aphid *Acyrthosiphon pisum* when challenged for 24, 48 and 72 h in the artificial diet.

## Discussion

Assays with sucking insects such as aphids can be tiresome and often suffer from instability and unreliability. For this reason, the technique for bioassay of aphids via artificial diet was improved. The advantages of the present method in comparison with previous techniques, that were reported by Mittler and Dadd (1964) and Auclair (1965), can be described as follows: it is an easy-to-use, practical and mechanically stable system. In addition, it facilitates observations of the probing activity and other aspects of the aphid's behavior within the cage. Finally, the escape of nymphs, even the smallest (first instar), has been eliminated, and also the contamination by fungi and bacteria was reduced. In addition a major advantage of this technique was the ability to test the efficacy of selected chemicals at doses lower than those needed in conventional screening bioassays, such as spraying or watering. The aphids in this technique received a much higher dose than would be expected from a surface spray because the chemical's effectiveness through time can be different for a surface spray as compared to being dissolved in solution. Further research is needed to clarify the relative utility of this method or an improved one (for instance with a combined oral and dermal exposure) for insecticides that are meant to be used as a topical spray applied to the surface as compared to those used as a systemic insecticide. In addition, for systemic products, the molecules need to be taken up from the surface in the plant sap stream, and this transport through the plant cells into the phloem sap (the actual feeding site of aphids) may cause a delay in toxicity. In some cases this can indeed lead to a rapid and high mortality with the artificial diet technique as the insecticide molecules reach the target site in the insect body very rapidly after direct oral uptake (sucking) of supplemented artificial diet as compared to other techniques such as spraying on plants. Regardless of these differences, we believe this strategy with artificial diet can be useful in screening the relative susceptibility of new chemicals against aphids under controlled conditions.

In this project the efficiency of the feeding apparatus was evaluated with biorational insecticides. In a first test, the toxicity of two novel selective feeding blockers, flonicamid and pymetrozine, as well as the neonicotinoid imidacloprid were bioassayed towards *A. pisum.* The strong insecticide activity obtained with flonicamid concurs with the only other report in aphids in the literature reported so far (Morita et al., 2007). The latter reported that the LC_50_ values ranged between 0.64 and 2.01 mg/1 when different plants (Japanese radish, eggplant, wheat, Chinese cabbage seedlings) were sprayed with flonicamid against different aphid species, *Myzus persicae, Aphis gossypii, Rhopalosiphum erysimi* and *Schizaphis graminum.* In addition, our experiments with flonicamid demonstrated that this novel compound rapidly inhibits the feeding behavior of aphids, i.e. within hours of treatment, without noticeable poisoning symptoms such as convulsion, and the aphids did not recover before dying.. This rapid activity is promising as it can contribute in controlling virus transmission. The results with the second feeding blocker pymetrozine also concur with recent findings. Foster et al. (2002) determined an LC_50_ value of 0.42 to 2.8 mg/1 towards first instars of *M. persicae* after 96 h, when tested at a range of 0.080 to 30 mg/1 in a leaf disk dipping bioassay. The respective LC_50_ values for pymetrozine were 2.3 and 27 mg/1 for the BC12-01 and WA19 clones of *Aphis pomi,* when tested in a leaf disk test. It should be marked that the latter values were somewhat higher than our results; however, toxicity may depend on the aphid species clone used (Lowery et al., 2006).

The data obtained in this project with imidacloprid confirm a strong activity of this neonicotinoid against sucking pest insects with an LC_50_ of 0.03 µg/ml against *A. pisum.* The high activity of neonicotinoids is also confirmed in other aphid species. Nauen and Elbert (1997) reported an LC_50_ of 0.07 µg/ml in artificial diet against a susceptible population of *M. persicae* and *Myzus nicotianae,* whereas for resistant aphids the LC_50_ was 14 mg/1. Lowery and Smirle (2003) determined an LC_50_ value of 0.064 mg/1 for imidacloprid when first instars of *A. pomi* were challenged for three days on treated apple leaf disks. In another leaf-dipping bioassay, the LC_50_ values ranged between 1.5–7.7 mg/1 for imidacloprid against different clones of *M. persicae* and *M. nicotianae* that were collected from different locations around the world (Devine et al., 1996). The latter experiments also showed that the sucking apparatus with artificial diet may be a useful tool in testing the susceptibility of different aphid strains/populations towards a selected insecticide under controlled conditions. We believe this strategy can be useful to monitor for resistance development, especially in populations from regions with high insecticide pressure.

Flufenoxuron is a benzoylurea type insecticide that inhibits chitin biosynthesis and cuticle formation in the nymphal stages of different pest insects (Ahn et al., 1993, Fisk et al., 1993). It is interesting to note that the toxicity of flufenoxuron against *A. pisum* was similar to the results obtained with azadirachtin. For comparison the respective LC_50_ values for larvae and eggs of codling moth, *Cydia pomonella,* were 9.9 and 5.4 mg/1 when apples were dipped in flufenoxuron.

The results obtained here with pyriproxyfen showed a much higher toxicity to first instar of *A. pisum* than 0.1% ZR-512 (hydroprene) by Kuhr and Cleere (1973) who documented the toxicity of ZR-512 towards seven species of aphids. They reported that when aphids were exposed for 72 h to host plants dipped in 0.1% ZR-512 mortality of first and second instar varied from 18% in turnip aphids, *Lipaphis erysimi,* to 73% in *A. pisum.* Similar results were reported by Liu and Chen (2001) in that the first three instars of *L. erysimi,* showed direct mortality and inhibition of growth and molting, and induced supernumerary-molted nymphs when exposed to pyriproxyfen at 50, 100 and 150 mg/1. In our tests with pyriproxyfen similar symptoms of mortality after blocked growth and molting were observed. Moreover, Richardson and Lagos (2007) recently showed that the survival of first instar nymphs of the soybean aphid, *Aphis glycines,* was 75% lower than in the controls when bioassayed with 50 mg/1 pyriproxyfen.

Halofenozide, a nonsteroidal ecdysteroid agonist, has been considered less lepidopteran selective than the other members of this group (Dhadialla et al. 1998). Results from this study showed that halofenozide can cause direct mortality and developmental disturbances due to inhibition of growth/molting. However, the potency was too low for commercial use. Nonetheless the feeding apparatus can be used as a miniature screening device for such novel more potent chemistries against sucking pest insects. Ecdysteroid agonists like halofenozide target the ecdysteroid receptor as an original site of action and this may provide a new strategy to counteract the wide resistance problems in aphids.

In the last decade it has been reported that azadirachtinbased insecticides have a deleterious effect against different aphid species in the laboratory and field trials (Lowery and Isman 1994; Ulrichs et al. 2001; dos Santos et al. 2004). In agreement with our results, Tang et al. (2002) reported an LC_50_ value of 4 mg/1 against second instar nymphs of the brown citrus aphid *Toxoptera citricida,* after 4 days of exposure to small seedlings previously dipped in Neemix. A similar result was found with first instars of *A. pisum* exposed for 7 days to plants treated with Margosan-O; the LC_50_ was 27.5 mg/1 azadirachtin (Stark and Rangus 1994). In addition, Hummel and Kleeberg (2002) found antifeeding effects by Neem-AzalPc (0.5% azadirachtin) in the bean aphid, *Apis fabae.* Ahmed et al. (2007) recorded 83% and 49% mortality in nymphs of *A. fabae* when challenged on plants treated with 0.2 and 0.05 µg/ml azadirachtin. The latter authors studied the systemic effects of neem products by keeping the plant seedlings in conical flasks including the nutrient solution mixed with Neemix and Neem-Azal T/S.

In conclusion, we have demonstrated an improved aphid feeding apparatus for the evaluation of insecticides with different modes of action and supplemented in small amounts via the liquid artificial diet. In most cases the data with the artificial diet confirmed results from leaf disk tests and plant experiments reported in the literature, demonstrating the usefulness of the feeding apparatus for screening insecticide activity against aphid pests. Interestingly, the present study demonstrated rapid and strong toxicity of a novel insecticide, flonicamid, and other biorational insecticides towards *A. pisum.*

## Abbreviations

**IGR**: insect growth regulator
